# Temporal and spatial variation in sex-specific abundance of the avian vampire fly (*Philornis downsi*)

**DOI:** 10.1007/s00436-021-07350-1

**Published:** 2021-11-20

**Authors:** Lauren K. Common, Petra Sumasgutner, Shane C. Sumasgutner, Diane Colombelli-Négrel, Rachael Y. Dudaniec, Sonia Kleindorfer

**Affiliations:** 1 College of Science and Engineering, Flinders University, Bedford Park, SA 5001 Australia; 2 Konrad Lorenz Research Center, Core Facility for Behaviour and Cognition, Department of Behavioural and Cognitive Biology, University of Vienna, Vienna, Austria; 3 Department of Biological Sciences, Macquarie University, Sydney, NSW 2109 Australia

**Keywords:** Range use, Ectoparasite, Invasive species, Galápagos Islands, *Philornis*

## Abstract

Understanding the range and behaviour of an invasive species is critical to identify key habitat areas to focus control efforts. Patterns of range use in parasites can differ temporally, across life stages and between sexes. The invasive avian vampire fly, *Philornis downsi*, spends the larval stage of its life within bird nests, feeding on developing nestlings and causing high levels of mortality and deformation. However, little is known of the ecology and behaviour of the non-parasitic adult fly life stage. Here, we document sex-specific temporal and spatial patterns of abundance of adult avian vampire flies during a single Darwin’s finch breeding season. We analyse fly trapping data collected across 7 weeks in the highlands (*N* = 405 flies) and lowlands (*N* = 12 flies) of Floreana Island (Galápagos). Lowland catches occurred later in the season, which supports the hypothesis that flies may migrate from the food-rich highlands to the food-poor lowlands once host breeding has commenced. Fly abundance was not correlated with host nesting density (oviposition site) but was correlated with distance to the agricultural zone (feeding site). We consistently caught more males closer to the agricultural zone and more females further away from the agricultural zone. These sex differences suggest that males may be defending or lekking at feeding sites in the agricultural zone for mating. This temporal and sex-specific habitat use of the avian vampire fly is relevant for developing targeted control methods and provides insight into the behavioural ecology of this introduced parasite on the Galápagos Archipelago.

**Supplementary Information:**

The online version contains supplementary material available at 10.1007/s00436-021-07350-1.

## Introduction

In an era of increasing human and animal global mobility, the proportion of invasive species is rapidly increasing, exacerbated by the effects of climate change, over-exploitation, pollution, and habitat fragmentation (Pelletier and Coltman 2018). Invasive parasites that pose risks to public health (González et al. 2017; Ruberanziza et al. 2019) or that negatively impact host species of conservation concern (Olson et al. 2013) warrant monitoring for informing control strategies. A single species may occupy and utilise different areas within its range across seasons, life stages, and between sexes (Bierzychudek and Eckhart 1988; Maxwell et al. 2019; Ruckstuhl and Neuhaus 2006). Understanding the distribution and behaviour of an introduced species is useful to identify seasonally or geospatially restricted habitat areas to focus control and management efforts (Escobar et al. 2019; Mathieu-Bégné et al. 2020; Raghavan et al. 2019; Woodworth et al. 2005).

In parasitic arthropods, studies have found selective spatial and temporal habitat use between the sexes (Papadopoulos et al. 2003; Sciarretta et al. 2018; Warburg and Yuval 1997; Wong and Jim 2018). In general, sexual conflict and sexual dimorphism have been shown to drive sex-specific distributions in arthropods (Foster and Soluk 2006; Romey and Wallace 2007; Stanley et al. 2018). Patterns of male and female abundance can differ due to sex-biased dispersal, with extremes where one sex disperses while the other is sedentary, which is especially relevant during range expansions or invasions (Beirinckx et al. 2006; Dudaniec et al. 2021; Miller and Inouye 2013). In other cases, females may aggregate together, away from areas of high male density, to avoid harassment from males, which is seen in systems with high costs to females from multiple mating (Roswell et al. 2019; Stanley et al. 2018; Stone 1995; Warburg and Yuval 1997). Understanding patterns of sex-specific distribution, location of oviposition, and feeding sites in invasive parasite populations is useful to control mating behaviours and frequencies, and to maximise the impact of targeted control interventions (Dunn and Hatcher 2015).

In resource-based mating, which is common in many insects, including parasitic insects (Dodson 1997; Preston-Mafham 2001; Warburg and Yuval 1997; Wilkinson and Johns 2005), males compete to guard a resource, such as a food or oviposition site, and mate with females that are attracted to the resource (Choe and Crespi 1997; Parker 1978). Resources must be predictable and defendable but uncommon enough to attract females. When resources are scattered or ubiquitous, swarm-based mating or mate searching systems, where males actively search for receptive mates, tend to prevail (Emlen and Oring 1977; Wilkinson and Johns 2005). Defended resources differ across species and are commonly food or oviposition sites (Preston-Mafham 2001; Wilkinson and Johns 2005). Males may lek near resources (Hendrichs et al. 1991; Warburg and Yuval 1997; Yuval 2005) or defend non-resource-based territories (Yeates and Dodson 1990) to attract and mate with females. Integrating information on the spatial and temporal distribution of invasive species, their mating systems, and monitoring of female population densities is therefore critical for the success of large-scale eradication programs (Enkerlin et al. 2017; Yamagishi et al. 1993).

The avian vampire fly (*Philornis downsi*, Dodge and Aitken, 1968) (Diptera: Muscidae) is a generalist invasive ectoparasite whose larvae consume the blood and tissue of developing birds across a range of host species (Dudaniec and Kleindorfer 2006; McNew and Clayton 2018). Introduced to the Galápagos Islands during the 1960s, avian vampire fly larvae were first discovered in Darwin’s finch nests in 1997 (Causton et al. 2006; Fessl et al. 2001). Since then, the fly has been detected on 14 islands across the archipelago (Fessl et al. 2018; Wiedenfeld et al. 2007). Adult avian vampire flies are non-parasitic and feed on fruit, nectar, and decaying vegetable matter (Fessl et al. 2018). However, their larvae are obligate parasites of nestlings, feeding both internally and externally on the host (Fessl et al. 2006; O’Connor et al. 2010a). In its invasive range, the avian vampire fly is highly virulent, causing severe in-nest mortality or alternatively, naris deformation in nestlings that persist into adulthood and thus affect song and foraging technique (Kleindorfer et al. 2019; Kleindorfer and Dudaniec 2016; Kleindorfer et al. in review). The effects of avian vampire fly parasitism, such as lowered body condition, naris deformation, and mortality, are of particular concern for declining populations of critically endangered Darwin’s finch species (Fessl et al. 2010; Lawson et al. 2017; O’Connor et al. 2010b).

Our understanding of adult avian vampire fly behaviour comes from genetic sources where multiple mating behaviour was established via larval sib-ship reconstructions (Dudaniec et al. 2010), or from video recordings at host nests that showed adult flies entering and leaving nests (Lincango et al. 2015; O’Connor et al. 2010a). Male and female avian vampire flies differ in their minimum longevity determined under laboratory rearing conditions (males ~ 188 days; females ~ 265 days) (Causton et al. 2019). The height at which adult flies were caught differed between the sexes on Floreana Island—females were more commonly caught at lowest and highest heights (2 m and 7 m) where there were fewer males (Kleindorfer et al. 2016). Wild avian vampire fly adults collected from Santa Cruz Island also have sex-specific microbiomes, which suggests the sexes may differ in diet and therefore foraging behaviour, perhaps due to different nutritional needs between sexes (Jose et al. 2021). Despite increasing knowledge on the avian vampire fly and its effects on hosts, we know little about where adult flies feed or mate. Mating behaviour has only been studied in a laboratory setting and has yet to be fully understood in the wild (Causton and Lahuatte pers. observation). In the laboratory, flies mate after being fed an enriched papaya diet and as well as a range of native and introduced plant species that have been offered to them, including the invasive blackberry (Causton and Lahuatte, pers. observation). As such, the agricultural zones of the inhabited islands may be an important area for avian vampire fly feeding because of the abundance of fruiting trees.

As the avian vampire fly is the greatest threat to the survival of all Galápagos land birds, it has been targeted for management and control (Causton et al. 2013). Therefore, it is critical to understand the various drivers of adult abundance and distribution. Host species nesting abundance may drive local avian vampire fly abundances, as adults may be attracted to nests for oviposition, and emerge from nests following development and pupation (Fessl et al. 2018). Although both larval and adult avian vampire fly populations occur ubiquitously across habitats (Causton et al. 2019; Dudaniec et al. 2007; Kleindorfer and Dudaniec 2016), intensity does differ across years and when accounting for host species (Kleindorfer and Dudaniec 2016).

It has been suggested that adult avian vampire fly populations may persist in highland refugia outside the host breeding season, where they can access agricultural crops and experience higher rainfall, and then disperse to lower, drier elevations once the host breeding season commences (Causton et al. 2013; Kleindorfer and Dudaniec 2016; Wiedenfeld et al. 2007). On Santa Cruz Island, catch rates of males decreased significantly across the non-breeding season in line with a shorter lifespan, while catch rates in females remained comparatively stable across the year (Causton et al. 2019). High rainfall was also shown to suppress daily catch rates of both male and female adult flies, likely due to decreased flight activity (Causton et al. 2019). Seasonal movements, key habitats, catch rates, and sex differences in habitat use in male and female avian vampire flies across islands are currently poorly understood. Due to the fly’s geographically widespread occurrence, identifying key sites and times of peak reproductive and dispersal activity within a Darwin’s finch breeding season is of special interest for the development of targeted control techniques, such as for mass release of biological control agents or sterile males.

In this study, we are interested in whether male and female avian vampire flies have different temporal or spatial distribution patterns that could be associated with different resource types (i.e. food resource = fruit from agricultural zone vs. reproductive resource = host nests) across two habitat types: humid, dense highlands, and dry lowlands. We quantify the number of male and female avian vampire flies captured in traps on Floreana Island, Galápagos, during the Darwin’s finch breeding season in 2020 and examine patterns of avian vampire fly abundance in relation to (a) date of trapping, (b) distance to the agricultural zone, and (c) the nesting density of Darwin’s finches within the highland and lowland study areas. We predict (a) female-biased sex ratio at the onset of breeding due to sex differences in minimum longevity and catch rates documented on Santa Cruz (Causton et al. 2019), (b) increased avian vampire fly abundance closer to the agricultural zone at the start of the breeding season, (c) increased avian vampire fly abundance, particularly female abundance as host nesting density increases, and (d) increased male abundance closer to the agricultural zone near higher densities of fruiting trees.

## Materials and methods

### Study site and species

**Fig. 1:**
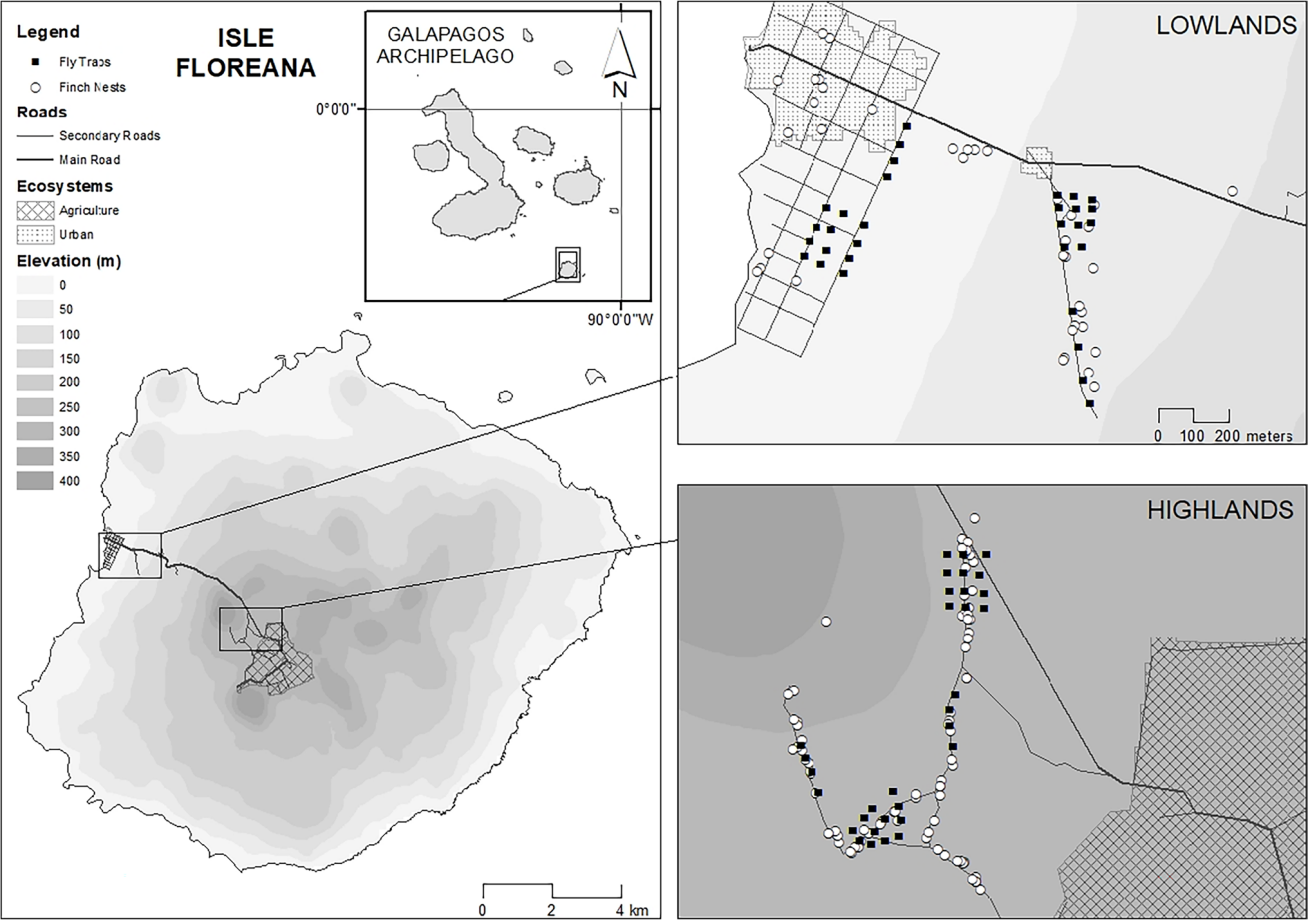
Map of Floreana Island, Galápagos Archipelago. McPhail trap locations are marked with black squares, location of Darwin’s finch nests monitored across the 2020 breeding season (small tree finch, medium tree finch, small ground finch and cactus finch) are marked with white dots

We collected adult avian vampire flies from traps on Floreana Island, Galápagos Archipelago, between January 19th and March 6th, 2020, during the Darwin’s finch breeding season. Trapping occurred in both the highlands and lowlands (Fig. 1). The highland site receives between 600 and 2300 mm of rain per year (Ben-Yosef et al. 2017; Charles Darwin Researcher Solanda Rea at Bella Vista; Galapagos Conservancy 2021). The highland site is a humid *Scalesia* forest at an elevation of 300–400 m asl located at the base of Cerro Pajas volcano (01°17′S, 090°27′W), and is adjacent to the agricultural zone (01°18′S, 090°26′W). The lowland site (01°16′S, 90°29′W) receives between 100 and 700 mm of rain per year (Charles Darwin Foundation Researcher Heinke Jäger at Puerto Ayora). The lowland is dominated by Palo Santo (*Bursera graveolens*) and Acacia (*Parkinsonia aculeata* and *Scutia spicata*) (Dvorak et al. 2017) with elevation of 0–150 m asl and is adjacent to the town of Puerto Velasco Ibarra (Fig. 1). On Floreana Island, human food production for the population (~ 110 people) occurs in the highland agricultural zone with only scattered fruiting trees near homes of individual families in the lowlands. Daily highland rainfall data were collected via satellite from CPC Global Unified Precipitation Data provided by NOAA/OAR/ESRL PSD, Boulder, CO, USA, downloaded from the Galápagos Vital Signs website (Galápagos Conservancy 2021).

The avian vampire fly is a myiasis-causing parasite whose free-living semi-hematophagous larvae feed on the developing nestlings of altricial birds (Dudaniec and Kleindorfer 2006; Fessl and Tebbich 2002). Avian vampire fly eggs are laid inside the host nests (O’Connor et al. 2010a) and once hatched, 1st instar larvae move to the nares and ear canals of newly hatched nestlings to feed on blood and tissue (Fessl et al. 2006). Second and third instar larvae generally reside in the base of the nest during the day, feeding internally (in nares) and externally on the nestlings at night (Fessl et al. 2006; O’Connor et al. 2010a). After 4–10 days of feeding, larvae pupate in a frothy cocoon in the base of the nest and emerge as adults after 7–18 days (Kleindorfer et al. 2014; Lahuatte et al. 2016). Adult flies feed on decaying vegetable matter including fruits and flowers (Fessl et al. 2018; Skidmore 1985), and can be attracted to baited traps using fruit juice lures (Lincango and Causton 2009).

### Avian vampire fly trapping

Adult vampire flies were collected using baited McPhail traps hung in trees (Causton et al. 2019; Lincango and Causton 2009). Traps were baited with 150 mL of liquid lure composed of 600-g ripe Hawaiian papaya, 75-g sugar, and 4 L of water, blended and fermented in the sun 3 days prior to use. Trapping occurred in the highland and lowland site. At each site, traps were placed within four study plots, each containing 12 traps separated by 50 m in a three by four trap lattice (Fig. 1). In addition, four traps were placed in two more study plots along a single transect each separated by 50 m (*N* = 32 traps per site, total *N* = 62 traps; Fig. 1). Traps were placed alternatively at 4 and 7 m high to capture potential sex ratio differences of flight height found previously by Kleindorfer et al. (2016). Bait lure was replaced and all specimens collected every 5 days. This was repeated nine times from January 19th to March 5th for a total of 563 trapping events. Collected flies were stored in 70% ethanol, identified, and sexed under a stereomicroscope following morphology described in Kleindorfer et al. (2016).

GPS coordinates were collected for each trap as they were deployed. Distance of each trap to the agricultural zone boundary was calculated using coordinate data. The host nesting density, i.e. the number of active Darwin’s finch nests per 200 m × 100 m study plot, was collected from our long-term nest monitoring protocol (see Kleindorfer et al. 2014), which occurred concurrently with trapping. Search effort for active nests within study plots was equal across highland and lowland sites. The host species monitored were the small ground finch (*Geospiza fuliginosa*), cactus finch (*Geospiza scandens*), small tree finch (*Camarhynchus parvulus*), medium tree finch (*C. pauper*), and the hybrid tree finch (*C. parvulus* × *C. pauper* as well as introgressed individuals). Each monitored host nest that was with eggs (incubation phase) or nestlings (feeding phase) within each study plot (100 m × 200 m) during each trapping period (5 days) was counted as an active nest, giving a nesting density of Darwin’s finch nests per plot for each trapping event.

### Mapping

Map figures were prepared using ArcMap 10.8.1 (ESRI 2011), with UTM 15S projection. Primary data obtained via ESRI Web Map included Ecosistemas Galápagos 2016, and Vías (roads) layers (ESRI 2017). The shoreline was obtained from NOAA Shoreline World Vector Shoreline (NOAA 2016; Wessel and Smith 1996) and the Digital Elevation Model (Souris 2018) was used to create elevation vectors at 50-m intervals for display purposes.

### Statistical analysis

All models were fitted in R version 4.0.0 (R Core Development Team 2020) using the packages ‘lme4’ (Bates et al. 2015), ‘car’ (Fox and Weisberg 2011), and ‘effects’ (Fox 2003). Results are presented as estimate ± standard error, unless otherwise stated. Total number of avian vampire flies (*N* = 417) across habitats (lowlands: *N* = 12; highlands: *N* = 405) was analysed using a generalised linear mixed model (GLMM) with negative binomial distribution to account for the non-normal distribution and over-dispersion of the count data. To incorporate the dependency among observations of the same trap, we used ‘trap ID’ as random intercept. To test for the effects of rainfall in highland avian vampire fly abundance, average daily rainfall was calculated per trapping event (5 days) for both sites. However, due to the strong correlation between average daily rainfall and Julian date (Pearson’s correlation test: rho =  − 0.71), rainfall was excluded from further analysis; patterns of rainfall for highlands are instead described in the results. The number of highland avian vampire flies caught in traps was analysed using negative binomial GLMM in relation to Julian date, distance to agricultural zone and host nest, the interaction term Julian date × distance to agricultural zone, with trap ID as a random intercept (*N* = 417) and a log link function. Corresponding analysis for male (*N* = 199) and female (*N* = 205) highland abundance in relation to Julian date, distance to agricultural zone, host nesting density, and number of the opposite sex caught in the same trap was analysed using GLMMs with negative binomial distribution, log link function, and trap ID as a random intercept. The proportion of male avian vampire flies, representing the sex ratio, was analysed separately for the highlands in relation to Julian date, distance to agricultural zone, and host nesting density using a GLM with the command bind (‘cbind’) function specifically designed to fit ratio data within the binomial family. The number of male avian vampire flies was the binomial denominator, quasibinomial (correct for overdispersion) distribution, and a logit link function. All quantitative variables were scaled (mean = 0 and standard deviation = 1) to bring variables into comparable scales and allow interpretation of the magnitude of all main effects (Grueber et al. 2011). Here, we report model effect sizes as estimate ± SE (summary function in ‘lme4’; Bates et al. 2015); *χ*^2^ and *p*-values from the ANOVA Table of Deviance using Type III *χ*^2^ tests (ANOVA function in the package ‘car’; Fox and Weisberg 2011).

## Results

**Fig. 2:**
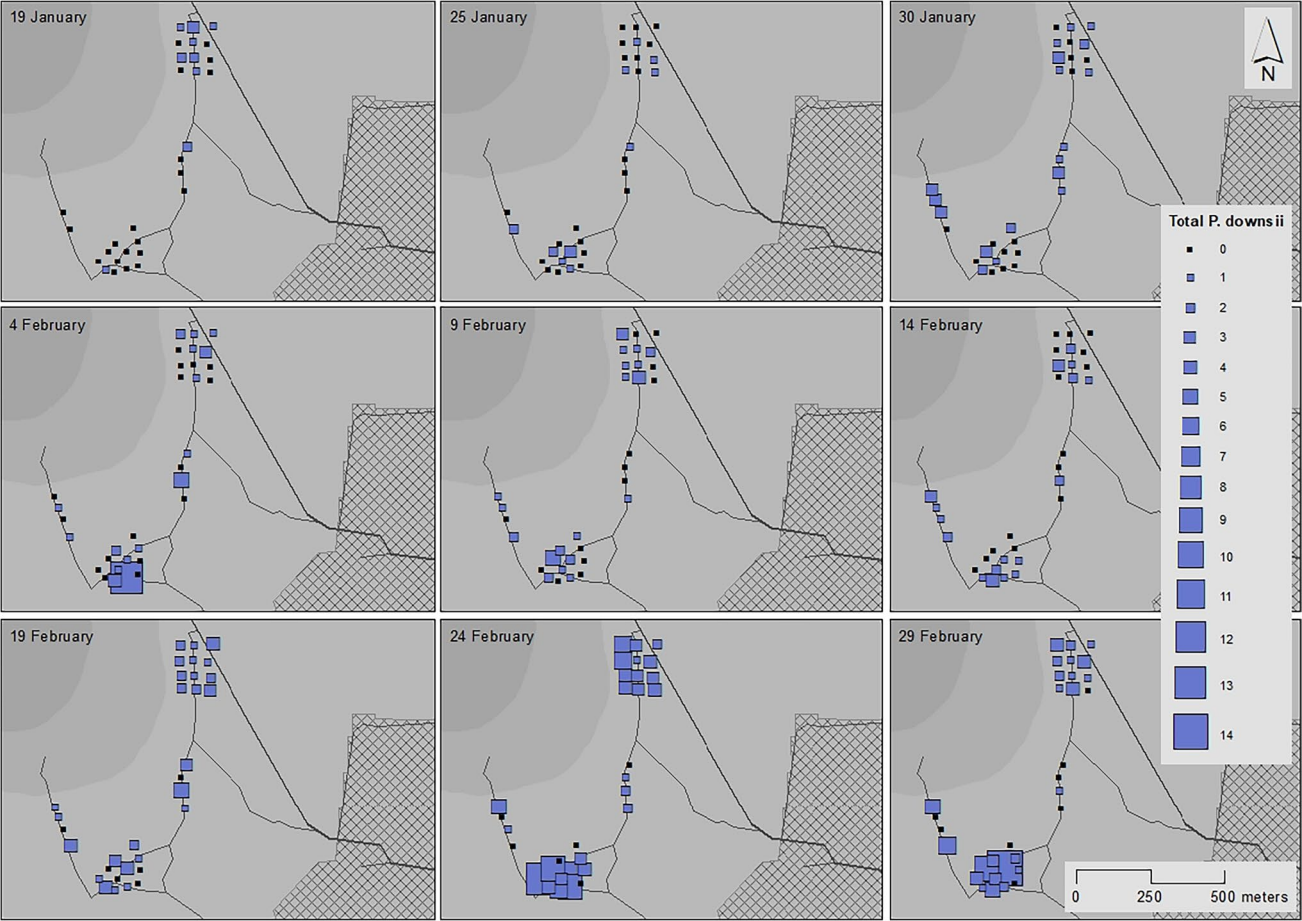
Avian vampire fly abundance per trapping event (top left of each frame indicates date of trap deployment, trapping events last 5 days) in the highlands of Floreana Island during the 2020 Darwin’s Finch breeding season (January 19th to March 5th)

The number of avian vampire flies was significantly higher in the highlands compared to the lowlands (GLMM, 3.49 ± 0.33, *p* < 0.001, Table 1; Fig. 2; Supplementary Fig. 1). Catch rates increased across the breeding season (0.57 ± 0.07, *p* < 0.001, Table 1). There was no effect of host nesting density on the number of flies caught (0.03 ± 0.07, *p* = 0.715, Table 1) and distance to agricultural zone was not included in the most parsimonious model. Trap ID accounted for 0.145 ± 0.38 of the variance in fly abundance. We caught a total of 12 avian vampire flies in the lowlands (0.003 males and 0.005 females per trap per day) and 405 in the highlands (0.135 males and 0.140 females per trap per day). At the onset of trapping (January 19th), which occurred before the onset of Darwin’s finch egg laying and nesting (approximately January 25th in highlands, January 31st in lowlands), we caught 13 males and 15 females from 59 traps in January in the highlands and no males or females from 62 traps in the lowlands. Average daily rainfall decreased across the study period (*t* =  − 17.037, df = 282, rho =  − 0.71, *p* < 0.001). During the first trapping period in the highlands (January 20th–25th), there was 31.9 mm of rain per day. At the end of the study period (February 29th–March 5th), rainfall was 2.0 mm per day.

**Table 1:** (a) Highland and lowland avian vampire flies (*Philornis downsi*) from McPhail traps collected during the Darwin’s finch breeding season. Generalized linear mixed model with negative binomial distribution for total number of adult avian vampire flies caught in relation to habitat, Julian date, and host nesting density. Trap ID was used as a random factor (variance = 0.145 ± 0.38). (b) Generalized linear model with quasibinomial distribution for avian vampire fly sex ratio in the highlands in relation to Julian date, distance to agricultural zone, and host nesting density. Sex ratio calculated as the proportion of male flies in relation to female flies. Avian vampire flies collected from McPhail traps on Floreana Island during the 2020 Darwin’s finch breeding season

(a) Total number of *Philornis downsi* (*N* = 417)	Estimate	SE	*z*-value	LR *χ*^2^	df	*P*-value
* Intercept*	* − 3.384*	*0.31*	* − 10.938*			< *0.001*
Habitat	3.492	0.33	10.582	111.99	1	< 0.001
Julian date	0.586	0.07	8.122	65.96	1	< 0.001
Host nesting density	0.027	0.07	0.365	0.13	1	0.089
(b) Highland *P. downsi* sex ratio (*N* = 405)	Estimate	SE	*t*-value	LR χ^2^	df	*P*-value
* Intercept*	* − 0.130*	*0.12*	* − 1.077*			*0.283*
Julian date	0.207	0.11	1.810	3.318	1	0.072
Distance to agricultural zone	− 0.516	0.11	− 4.731	23.686	1	< 0.001
Host nesting density	− 0.051	0.12	− 0.415	0.173	1	0.678

Dispersion parameter for negative binomial model (a) taken to be 1.981 for quasibinomial model (b) taken to be 0.938

### Lowlands

Flies were not collected in the lowlands until the fourth replicate of trapping (February 5th–February 10th; Supplementary Fig. 1), despite equal trapping effort across the season and across habitats. On the contrary, in the highlands, flies were collected in the first replicate of trapping (January 20th–January 25th; Fig. 2). Due to the small sample size of flies collected in the lowlands (*N* = 12) and low statistical power, we are unable to analyse the effects of host nesting density on adult avian vampire fly abundance or change in sex ratio across the season in the lowlands.

### Highlands

**Fig. 3:**
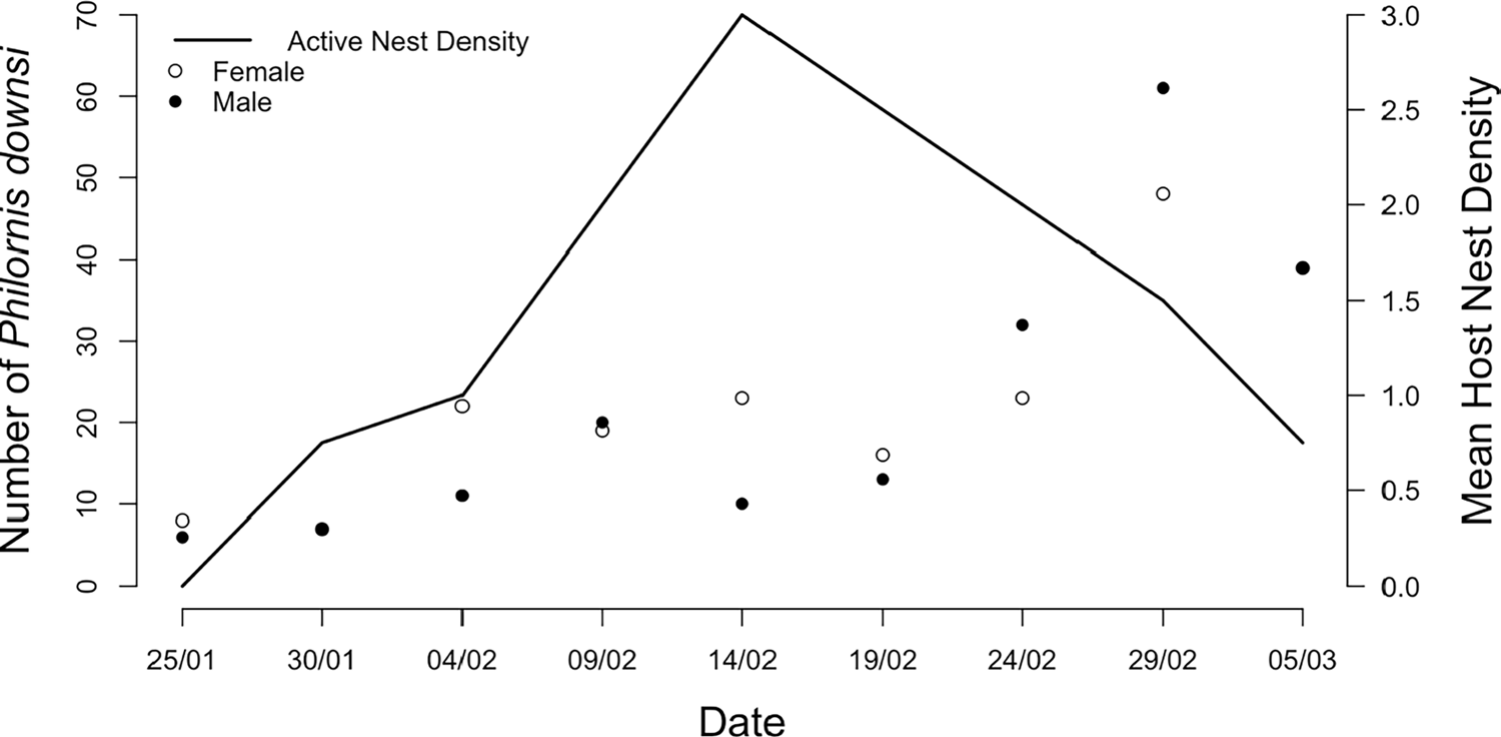
Highland number of male and female avian vampire flies (*Philornis downsi*) across date of collection (trapping replicate duration 5 days) with mean host nesting density per study plot (200 m × 100 m). Open circles represent the number of female avian vampire flies caught in the highlands during a trapping event, closed circles represent the number of male avian vampire flies caught across the same time period. Avian vampire flies collected from McPhail traps in the highlands of Floreana Island in 2020 during the Darwin’s finch breeding season (January 19th to March 5th)

Highland avian vampire fly abundance (*N* = 405) increased across the breeding season (GLMM, 0.57 ± 0.07, *p* < 0.001, Table 2, Fig. 2). There was no effect of distance to the agricultural zone (0.09 ± 0.01, *p* = 0.370, Table 2) or host nesting density (0.04 ± 0.08, *p* = 0.607, Table 2) on the overall abundance in the highlands. There was also no interaction effect between trapping date and distance to the agricultural zone (− 0.08 ± 0.07, *p* = 0.273). Trap ID accounted for 0.16 ± 0.40 of the variance in highland abundance. The sex ratio did not change significantly across the breeding season (GLM, 0.21 ± 0.12, *p* = 0.072, Table 1). There was no effect of host nesting density (*N* = 16 total active nests in highland study plots across breeding season, Fig. 3) on sex ratio when accounting for date of capture (− 0.05 ± 0.12, *p* = 0.679, Table 1; Fig. 3). The sex ratio was highly skewed towards males closer to the agricultural zone (− 0.51 ± 0.11, *p* < 0.001, Table 1) and skewed towards females further from the agricultural zone.

**Table 2:** Highland avian vampire flies (*Philornis downsi*) from McPhail traps collected in 2020 during the Darwin’s finch breeding season from Jan to Mar. Generalised linear mixed model for (a) total number of adult avian vampire flies in relation to Julian date, distance to agricultural zone, elevation, and host nesting density (random factor trap ID variance = 0.16 ± 0.40); (b) number of male avian vampire flies in relation to Julian date, distance to agricultural zone, host nesting density, and number of females (trap ID variance = 0.06 ± 0.25); (c) number of female avian vampire flies in relation to Julian date, distance to agricultural zone, host nesting density, and number of males (trap ID variance = 3.4 × 10^−9^ ± 5.8 × 10^−5^). N is the raw number of flies caught

a) Total number of *Philornis downsi* (*N* = 405)	Estimate	SE	*z*-value	LR *χ*^2^	df	*P*-value
* Intercept*	*0.106*	*0.11*	*1.005*			*0.315*
Julian date	0.562	0.07	7.627	58.167	1	< 0.001
Distance to agricultural zone	0.065	0.10	0.648	0.420	1	0.517
Host nesting density	0.042	0.08	0.076	0.300	1	0.584
Julian date × Distance to agricultural zone	− 0.081	0.07	− 1.096	1.201	1	0.273
b) Male *Philornis downsi* (*N* = 199)	Estimate	SE	*z*-value	LR χ^2^	df	*P*-value
* Intercept*	* − 0.735*	*0.12*	* − 6.260*			< *0.001*
Julian date	0.544	0.10	5.420	29.373	1	< 0.001
Distance to agricultural zone	− 0.267	0.10	− 2.645	6.994	1	0.008
Host nesting density	0.045	0.10	0.449	0.202	1	0.653
Number of females	0.373	0.07	5.238	27.440	1	< 0.001
c) Female *Philornis downsi* (*N* = 205)	Estimate	SE	*z*-value	LR χ^2^	df	*P*-value
* Intercept*	− *0.548*	*0.09*	− *6.244*			< *0.001*
Julian date	0.283	0.09	3.269	10.687	1	< 0.001
Distance to agricultural zone	0.380	0.08	4.676	21.862	1	< 0.001
Host nesting density	0.039	0.08	0.464	0.215	1	0.643
Number of males	0.372	0.06	6.582	43.329	1	< 0.001

Dispersion parameter for negative binomial model (a) taken to be 1.925; (b) taken to be 2.455; (c) taken to be 5.966

Examining abundance patterns in each sex in the highlands separately, male avian vampire fly abundance (*N* = 199) increased across the breeding season (GLMM, 0.54 ± 0.10, *p* < 0.001, Table 2), and increased closer to the agricultural zone (− 0.27 ± 0.10, *p* = 0.008, 2), with no effect of host nesting density (0.04 ± 0.10, *p* = 0.653, Table 2). There was a positive association between the number of male flies and female flies collected in the same trap (0.37 ± 0.07, *p* < 0.001, Table 2), as the number of female flies increased, so did the number of male flies. Trap ID accounted for 0.06 ± 0.25 of the variance in male fly abundance. Female avian vampire fly abundance (*N* = 205) increased across the breeding season (GLMM, 0.28 ± 0.09, *p* < 0.001, Table 2), decreased closer to the agricultural zone (0.38 ± 0.08, *p* < 0.001, Table 2), and did not increase in relation to host nesting density (0.04 ± 0.08, *p* = 0.643, Table 2; Fig. 3). Trap ID only accounted for 3.4 × 10^−9^ ± 5.8 × 10^−5^ of the variance in female abundance.

## Discussion

Understanding sex-specific range use, key resources, and habitat use by populations is critical for developing effective control techniques for invasive species. We found significant differences in temporal patterns of abundance of the invasive avian vampire fly across the Darwin’s finch breeding season and two habitat types on Floreana Island. The number of flies caught in traps increased significantly across the breeding season (January–March), which might be due to increasing numbers of adult flies emerging from host nests towards the end of the host breeding season. The prevalence of *P. downsi* in monitored nests with nestlings was 100%; however, if the nests failed during incubation, no avian vampire fly larvae were found. Contrary to our prediction, there was no effect of host nesting density on overall abundance or sex-specific abundance. Our data suggest that avian vampire flies may use the highland *Scalesia* and nearby agricultural zone as a refugium during the non-breeding season. Adult flies were collected in the highlands during the first trapping event but were not collected in the lowlands until 20 days into trapping. Avian vampire flies may have dispersed from the humid highlands to the arid lowlands as host breeding increased, as there is evidence that adult flies are capable of dispersing large distances (Fessl et al. 2018). We did not find support for our predictions of a female-biased sex-ratio or increased abundance near the agricultural zone at the onset of the breeding season. This contrasts with previous research that found males have a shorter lifespan and are unlikely to survive to the next year’s breeding season, resulting in a female-biased sex ratio (Causton et al. 2019). Although the sex ratio remained stable across habitats (highlands vs lowlands) and time, we caught more male avian vampire flies closer to the agricultural zone, with females showing the opposite pattern. Our results highlight the importance of the highland habitat, on Floreana Island, as a key location to plan control measures for the avian vampire fly.

The sex differences in catch numbers close to the agricultural zone raise questions about the mating behaviour of the avian vampire fly. It is possible, though untested, that males near the agricultural zone may be guarding food resources or lekking near fruits to attract females. In many species that display resource-based mating, males defend oviposition sites, with both mating and oviposition occurring at the guarded site (Preston-Mafham 2001; Warburg and Yuval 1997; Wilkinson and Johns 2005). This may not be a preferred option for male avian vampire flies, due to the presence of the incubating or brooding female at the nest (Kleindorfer et al. in review). There is video evidence that avian vampire flies wait outside the host nest, only entering once the nest is unattended, and leaving once the female returns (Lincango et al. 2015; O’Connor et al. 2010a). There may be a risk of predation by the insectivorous bird if a fly enters or mates near an occupied nest, making the host nest a less attractive ‘mate attraction’ resource, especially when considering that the length of mating reported under laboratory conditions is often between 5 and 7 min (Causton and Lahuatte et al., pers. comms.). Sex differences in dispersal or climatic tolerance (Enriquez and Colinet 2017; Lyons et al. 2014; Miller and Inouye 2013) are other explanations for the possible differences in spatial patterns we observed. The proximity to the agricultural zone stands out as a key element that warrants further study and points to possible differences in nutritional needs between the sexes or mating behaviour. A key factor known to drive sex-specific ranges is male harassment, which may operate in conjunction with or independently of the mating system (Stanley et al. 2018; Stone 1995). Male harassment, and its associated costs to females, has previously been suggested as one possible explanation for sex-specific micro-habitat use in the avian vampire fly (Kleindorfer et al. 2016), though it is still untested. Due to the limitations of this study, we are unable to determine the cause of the sex-specific distribution in avian vampire fly on Floreana Island during 2020. Future research could test ideas to disentangle the potential role of mating system and foraging ecology in avian vampire flies in relation to proximity to fruiting trees in the agricultural zone.

Although we did not find a correlation between host nesting density and number of avian vampire flies, there may be a time lag between nest termination and fly emergence, and therefore an increase in fly abundance. Due to the restricted sampling period of this study, we do not know how the abundance of the avian vampire fly changes after host breeding has finished on Floreana. On Santa Cruz, female catch rates remained approximately stable across the non-breeding season, with male catch rates decreasing between breeding seasons (Causton et al. 2019). It is possible that the fly population decreases slowly after the breeding season as adult flies die off. This is in contrast to the rapid drop-off in nesting density seen in this study, which may be why we did not find a relationship between fly abundance and host density; however, this remains to be tested. Other host-parasite systems display a parallel pattern of density in host and parasite populations (Byers et al. 2008; Oorebeek and Kleindorfer 2008; Young et al. 2015); however, this pattern is not universal (Cardon et al. 2011). Extending the trapping duration outside of the host breeding season can further explore the sex-specific population dynamics on Floreana Island in relation to host nesting density.

The marked habitat differences in number of flies caught between highlands (*N* = 405) and lowlands (*N* = 12) could be explained by rainfall, host density, or number of fruiting trees, whereby our patterns stand in marked contrast to those found on Santa Cruz Island (Causton et al. 2019). On Santa Cruz Island, catch rates of avian vampire fly adults, in particular female flies, was higher in lowland sites compared to highland sites (Causton et al. 2019). Differences in catch rates between the sexes in these two studies—0.08 females vs 0.08 males per trap per day in this study compared to 0.25 females vs 0.08 males per trap per day in Causton et al. (2019)—are likely due to the differences in sampling duration (6 weeks versus 1 year). It is also notable that the Santa Cruz lowland sites were sampled near a dense urban area with higher human population and potential access to fruiting trees, fresh water, and human food (INEC 2015). Causton et al. (2019) trapped adults across the entire year and found lower catch rates for male avian vampire flies during the host non-breeding season. In contrast to Causton et al. (2019), we did not catch fewer males at the start of the host breeding season. The number of birds is generally lower in the lowlands than in the highlands on both Santa Cruz and Floreana Islands (Dvorak et al. 2012; Dvorak et al. 2017). In terms of nesting, across the same time period, monitoring effort, and area, we encountered five lowland nests with eggs and 16 highland nests with eggs (Kleindorfer et al. unpublished). While the summary data suggest that host nesting density could explain the different trapping success in lowlands versus highlands, the host nesting density did not predict the number of adult flies caught in highland traps, and so we reject this explanation. We suggest that rainfall, human population, and fruiting trees may be more important factors for avian vampire fly abundance.

Although our results are based on one season of data, the clear patterns combined with conservation urgency call for future research and targeted intervention. For example, the Sterile Insect Technique (Hendrichs et al. 2002), whereby large numbers of sterile insects that produce no offspring are released, could be targeted to high male density areas. Utilising mating or attractant pheromones to disrupt mating (Carde and Minks 1995) can be deployed where mating most commonly occurs. Future research should extend trapping into the non-breeding season and within the agricultural zone at particular fruiting trees to determine patterns of migration across habitat types (Midgarden et al. 2014). Sex-specific population control could be effective in managing and eradicating invasive populations, especially in populations that display sex segregation (Papathanos et al. 2014). If the avian vampire fly is restricted to the highlands and agricultural zone during the non-breeding season, with males primarily distributed in the agricultural zone, this could be a critical area for male population suppression (Hendrichs et al. 2005). Male annihilation techniques, such as those used to decrease fruit fly populations, could be used during the non-breeding season to decrease male density, which can be used alone or in conjunction with sterile insect release (Vargas et al. 2014). With promising developments on survival rates for reared avian vampire fly larvae in a laboratory setting (Lahuatte et al. 2016), the potential for sterile insect breeding and release is increasing, although more research is needed.

Invasive parasites often pose challenges to biodiversity, but they also represent a chance to learn about novel host-parasite systems under new evolutionary selection regimes (Allendorf and Lundquist 2003; Sakai et al. 2001). In an emerging host-parasite system on Floreana that was likely established after 1960 (Kleindorfer and Sulloway 2016), and has been studied since 2004 (Kleindorfer et al. 2014), we find differences between habitats in numbers of adult flies and sex ratio in relation to proximity to the agricultural zone and across the breeding season. In neither sex did host nesting density predict number of flies caught, but we caught more males close to the agricultural zone at the onset of the breeding season. Intriguingly, the patterns we found are markedly different from those on Santa Cruz Island (Causton et al. 2019), where the number of flies caught was higher in the lowlands than in the highlands and a female-biased sex ratio at the onset of the host breeding season occurred. If the avian vampire fly populations are inhabiting different areas on different islands, this could pose additional challenges for biological control. Future work should extend trapping into the non-breeding season and assess long-term abundance changes in host and parasite abundances across years. Understanding of the movements and populations of both sexes of the avian vampire fly, such as the findings of this study, can inform island-specific targeted control, particularly relevant to control techniques that manipulate breeding behaviour such as the Sterile Insect Technique and pheromone-based mating disruption. This information, as well as the possible occurrence of lag effects of parasite abundance, would further inform the optimal timing and distribution of deploying control efforts. Further research into the spatial and temporal behaviour and ecology of the avian vampire fly in relation to island is critical to untangle the various drivers of adult populations in its invasive range.

## Supplementary Information

Below is the link to the electronic supplementary material.

Supplementary file1 (DOCX 381 KB)

## Data Availability

Data reported in this study will be uploaded to Dryad Digital Repository upon acceptance.

## Declarations

### Permits

Permission to conduct this study was given by the Galápagos National Park and Charles Darwin Research Center, permit no. PC-02–20: Invasivos e invasivos: estableciendo la línea base para evaluar la iniciativa de control de roedores en Floreana sobre la densidad y comportamiento de anidación de pinzones de Darwin y el parasitismo por *Philornis downsi*, and Flinders University, permit no. E480/19.
