# Supplementary material for: Temporal and spatial variation in sex-specific abundance of the avian vampire fly (Philornis downsi)
Source: Parasitol Res. 2021 Nov 20;121(1):63–74. doi: 10.1007/s00436-021-07350-1 (PMC8748338; doi:10.1007/s00436-021-07350-1)

**Supplementary Material**

**Fig. 1:** Avian vampire fly abundance per trapping event (top right of each frame indicates date of trap deployment, trapping events last 5 days) in the lowlands of Floreana Island during the 2020 Darwin’s Finch breeding season (January 19^th^ to March 5^th^).


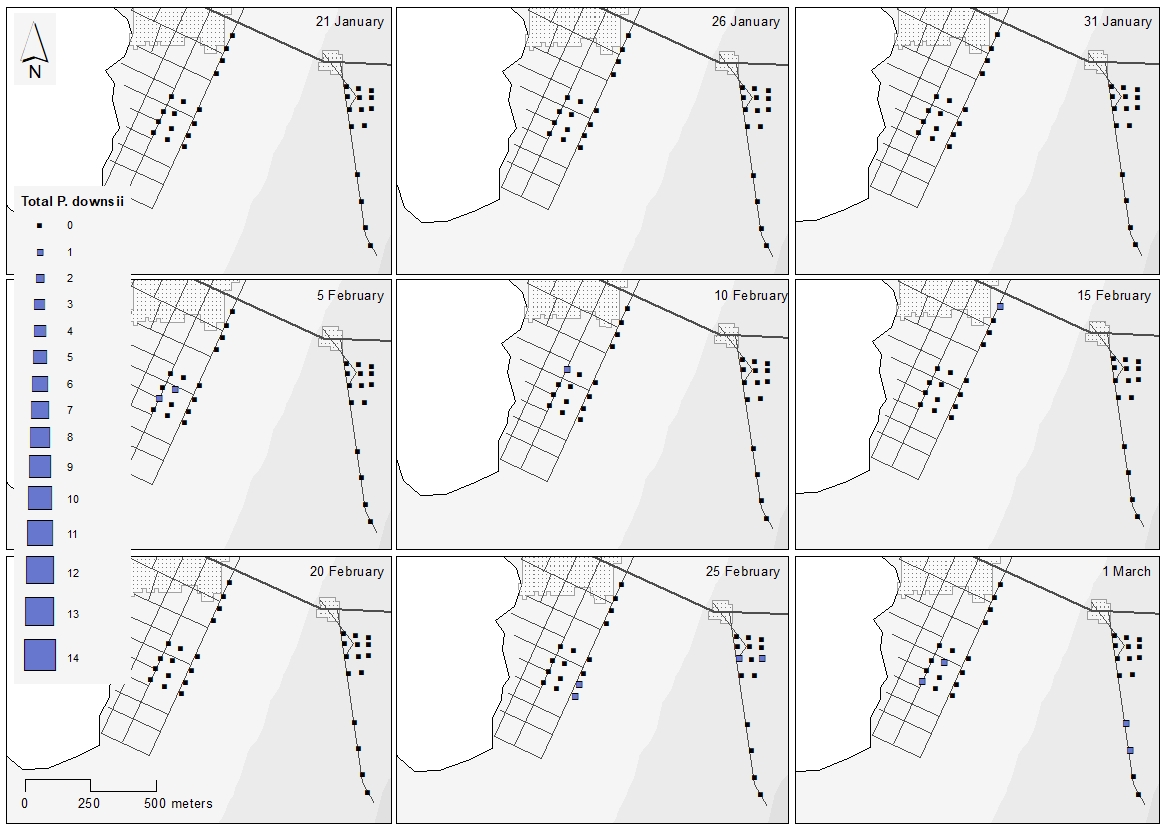

Supplement: Supplementary file 1 — Supplementary file1 (DOCX 381 KB) [file 436_2021_7350_MOESM1_ESM.docx]
